# Utility of Shear Wave Elastography for the Diagnosis of Plantar Fasciitis: Comparison Between Symptomatic and Asymptomatic Sides in Unilaterally Affected Patients

**DOI:** 10.7759/cureus.60231

**Published:** 2024-05-13

**Authors:** Rajani Thakur, Sundeep Kund Reddy Aluka, Rama Srikanth, Syed Maqsood Hussain

**Affiliations:** 1 Radiology and Imageology, Nizam's Institute of Medical Sciences, Hyderabad, IND; 2 Orthopaedics, Nizam's Institute of Medical Sciences, Hyderabad, IND

**Keywords:** swe in musculoskeletal imaging, swe, ase, shear wave elastography in plantar fasciitis, elastography in plantar fascitis

## Abstract

Introduction: Plantar fasciitis (PF) can cause pain in the heel, which can affect everyday activities. While it often resolves on its own, diagnosing PF to rule out other hind foot conditions by imaging modality in cases of recurrence can be difficult. Methods such as MRI and ultrasonography are helpful, but the use of elastography, specifically shear wave elastography (SWE), as a tool for diagnosing PF is being studied.

Methodology: This comparative observational study included patients over 18 years presenting with unilateral hind foot pain who were investigated using SWE. Exclusions comprised those who were bilaterally affected and with foot deformities, trauma history, or prior injection therapy. Patients' AOFAS Ankle-Hindfoot Scores were assessed along with visual analog scale (VAS) scores, followed by SWE examination of both heels.

Results: The study found no significant difference in the plantar fascia thickness between affected and unaffected sides, with a mean thickness of 4.3±0.8mm and 5.1±0.6mm, respectively. Shear wave velocity (SWV) was lower on the affected side, indicating reduced stiffness compared to the unaffected side. The Spearman rank test revealed strong direct correlations between SWV and both the VAS and HF-AOFAS scores on the affected side.

Conclusion: The study observed that SWE enhances B-mode ultrasonography in detecting early PF even with normal plantar fascia thickness, offering a user-independent and reliable tool for treatment monitoring and correlation with functional and pain scores. Further research with larger populations can aid in developing a clinico-radiological classification system for PF, improving prognostication and treatment guidance.

## Introduction

Plantar fasciitis (PF) is the common cause of hindfoot pain and has a prevalence of 10% worldwide [1]. It debilitates the daily routine of the affected, particularly in the early morning hours or after a brief period of rest, before walking. In 80% of people, it is self-resolving, but debilitating daily activities make them seek medical advice [2]. Etiopathogenesis remains multifactorial, and most of the time, it is difficult in clinical scenarios to analyze the cause unless there are obvious deformities associated or there is gastrocnemius tightness. Histological analysis of PF revealed more degenerative changes (fasciosis) than inflammation (fasciitis) [3]. Differential diagnoses of PF include fat pad contusion or atrophy, stress fractures, plantar fibromatosis or Ledderhose disease, and nerve entrapments such as tarsal tunnel syndrome or Baxter nerve entrapment [4]. Patient dissatisfaction is more with conservative trials of management of PF and recurrence of pain is more frustrating. To avoid long-term suffering due to a wrong diagnosis, there is a need for an ideal investigation to support the diagnosis of PF. Draghi et al. have outlined the imaging features of PF in their review and recommend imaging like MRI and ultrasonography to rule out other differential conditions like nerve entrapment, plantar fibromatosis, or Ledderhose disease [5]. Enthesopathy findings such as thickening of the PF; intrasubstance areas of intermediate signal on T1-weighted sequences and increased signal on fluid-sensitive sequences; edema in the adjacent soft tissue; bone marrow edema of the calcaneal attachment of the PF are commonly observed in MR imaging [5]. Sonographic findings (Gray-scale imaging, B-US) indicative of plantar fasciitis include loss of fibrillar structure, thickening over 4 mm, perifascial collections, and any calcifications within the plantar fascia [6]. Although sonography (B-US) can differentiate PF from other hind foot disorders, its ability to detect early cases of PF and to prognosticate the severity of the disease is limited based on morphological characteristics alone.

Axial strain elastography (ASE) and shear wave elastography (SWE) have been extensively reviewed in patients with PF in recent years [7]. The ASE technique utilizes a probe to externally compress tissue, resulting in semi-quantitative and user-dependent information regarding tissue elasticity. Conversely, SWE generates an impulse with a probe and precisely measures elastographic parameters in Kilopascal (kPa, m/s) as an elastic module (Young's modulus, kPa) or as shear wave speed progression (m/s) [7]. Since SWE is user-independent, it can be a better tool than other elastography measures in cases of PF. The purpose of this study was to compare SWE measurements of the symptomatic and asymptomatic sides in unilaterally affected patients. By analyzing objective SWE parameters in the Indian population, the study aimed to gain new insights into the changes in the plantar fascia associated with PF. These insights could prove helpful in diagnosing cases that are otherwise unclear.

## Materials and methods

This is a comparative observational study among patients presenting with unilateral PF to the Department of Orthopedics at our institute (Nizam's Institute of Medical Sciences, Hyderabad) from April 2021 to April 2023. The study was conducted in accordance with the Declaration of Helsinki protocol after the included patients consented to participate. All patients aged above 18 and who presented with unilateral hind foot pain were investigated with SWE. Patients with concurrent or previous bilateral affection, a history of ankle and foot deformity or trauma or previous surgery, and previous injection therapy (platelet-rich plasma, steroid, growth factor concentrate) were excluded from the study. Orthopedicians evaluating the patient have examined the patients in detail and noted that all the patients had pain at the insertion of plantar fascia, that increased with great toe dorsiflexion (Windlass Test). None of the patients had any stress fractures and there was no minimum duration period necessary to exclude from the study. All the included patients' VAS and AOFAS Ankle-Hindfoot Score [8] were assessed and they were subjected to SWE to both heels. 

**Figure 1:**
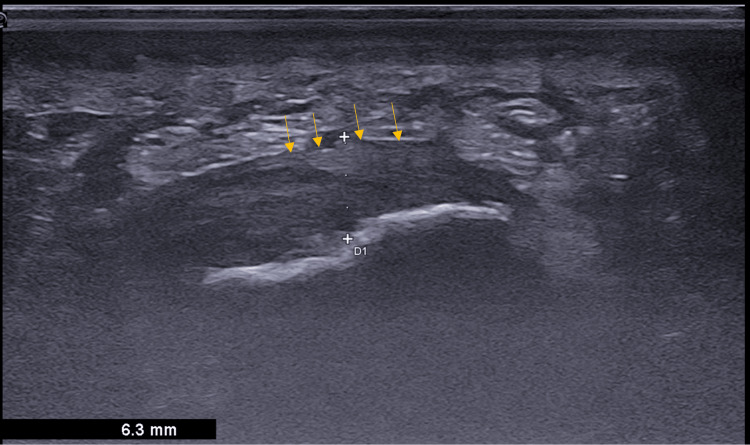
Sagittal B-mode ultrasound image of a 38-year-old female patient with plantar fasciitis shows hypoechoic, thickened plantar fascia measuring 6.3 mm (arrow showing the region of interest) with loss of the fibrillar pattern.

**Figure 2:**
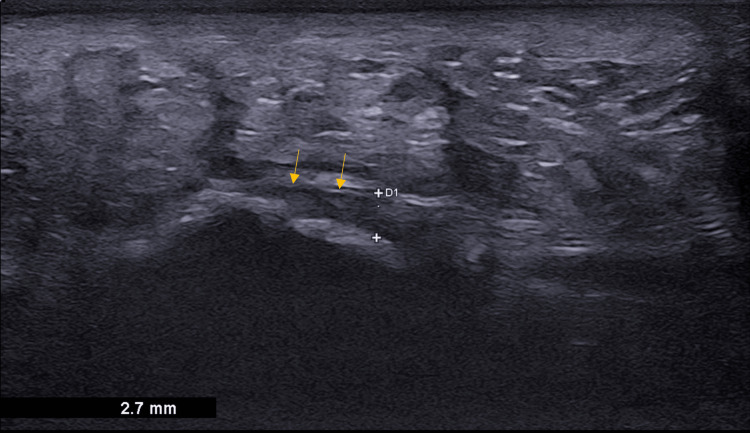
Normal plantar fascia: Sagittal B-mode ultrasound shows normal plantar fascia with the maintained fibrillar pattern and normal thickness measuring 2.7 mm (arrow showing the region of interest).

All SWE examinations were done using a 4-15 MHz probe on MyLab™9 eXP ultrasound system (Make: Esaote, Genova, Italy). All measurements were done with patients in a prone position and foot dangling from the edge of the couch in a relaxed fashion. A longitudinal B-mode scan was done to measure the thickness of plantar fascia on the affected and unaffected sides (Figure 1 and Figure 2).

**Figure 3:**
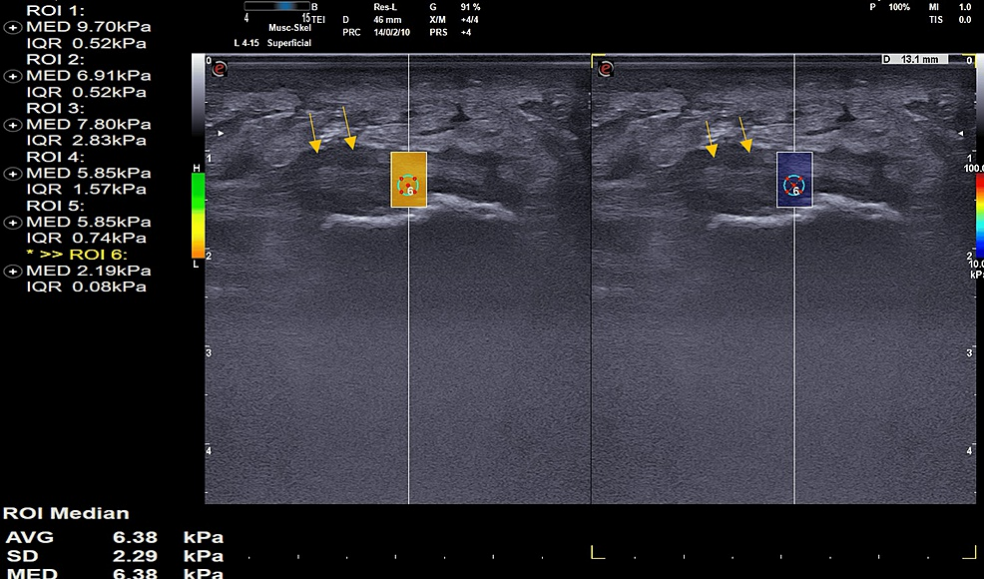
Plantar fasciitis: Sagittal B-mode ultrasound shows thickened, hypoechoic plantar fascia with loss of the fibrillar pattern (arrow showing the region of interest). The longitudinal SWE ultrasound image of a 38-year-old patient with plantar fasciitis demonstrates a stiffness of 6.38 Kpa. Kpa: Kilo Pascals

**Figure 4:**
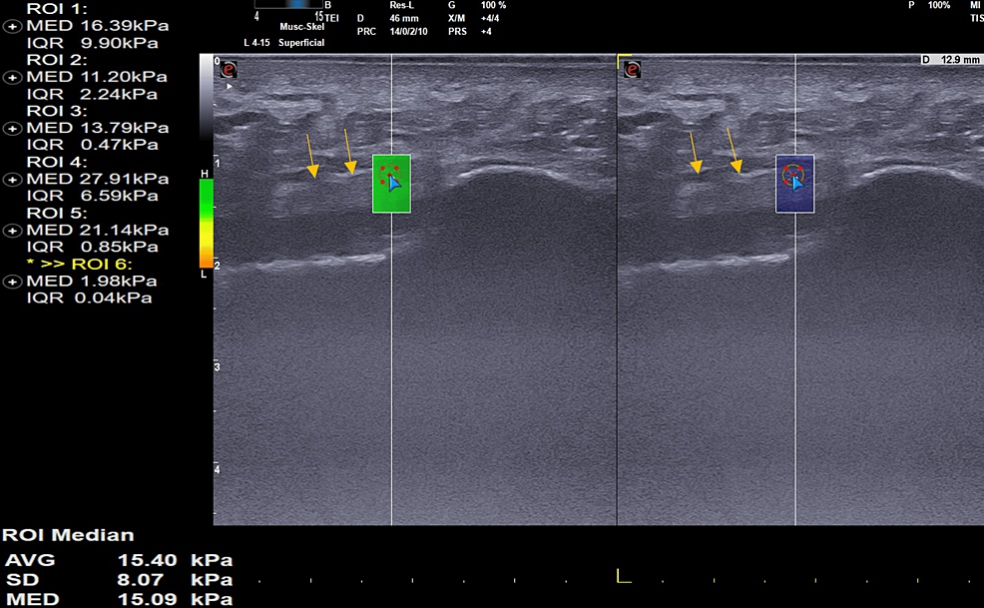
Normal plantar fascia: Sagittal B-mode ultrasound shows normal plantar fascia with the maintained fibrillar pattern (arrow showing the region of interest). The longitudinal SWE ultrasound image of a 40-year-old healthy volunteer with normal plantar fascia demonstrates a stiffness of 15.09 Kpa. SWE: Shear wave elasticity; Kpa: Kilo Pascals

The SWE measurement window was 2 cm^2^. To perform quantitative SWE measurements, a region of interest (ROI) with a diameter of 2 mm was placed at the thickest area in the plantar fascia. The obtained SWE information assessed Young's modulus (kPa) and shear wave speed (m/s). Each plantar fascia was systematically analyzed in longitudinal plane at 1 cm below calcaneal insertion and an average of three readings was noted down (Figure 3 and Figure 4).

The study used IBM SPSS Statistics for Windows, Version 24 (Released 2016; IBM Corp., Armonk, New York, United States) for statistical analysis. The results were shown as mean and range. 

## Results

A total of 27 patients with unilateral plantar fasciitis satisfying all inclusion and exclusion criteria were included in the study. Demographic data and pain characteristics of the study group are shown in Table 1. There was no significant difference in the affected side in the study population (p>0.05). 

**Table 1:** Plantar fascia thickness and SWV among symptomatic and asymptomatic groups.

	Symptomatic Side	Asymptomatic	p-value
Plantar Fascia Thickness in mm	5.5± 0.7mm	4.3 ± 0.8mm	p>0.05
Shear Wave Velocity (SWV) in m/s	4.4 ± 0.7	6.8 ± 1.2	p=0.003

We found that there was no significant difference in the thickness of plantar fascia on the affected side compared to the unaffected side. The mean thickness of PF was 4.3 ± 0.8mm on the unaffected side and about 5.5± 0.7 mm on the affected side and was not statistically significant (p>0.05). SWV on the affected side had lower values indicating lesser stiffness compared to the unaffected side which had higher SWV (Table 2). The Spearman rank test was used to assess the correlation between SWV and the clinical scores on the affected side and it was found that there was a strong direct correlation between SWV and the VAS score (r = − 0.40; p = 0.001) and between SWV and the HF-AOFAS score (r = 0.59; p = 0.012).

**Table 2:** Demographic and pain characteristics of the study group. Data are shown as mean and standard deviation. VAS: Visual analog scale

	Age (years)	Sex	Side	BMI	VAS	HF-AOFAS
Symptomatic Side	40.9 ± 13.5	Male (n= 11) Female (n=16)	Left = 15 ; Right = 12	26.06 ± 4.8	7.2 ± 1.3	69.8 ± 9.8
Asymptomatic Side			Right = 12 ; Left = 15		0	100

## Discussion

B-mode ultrasonography is a cheaper and easily available investigation tool for PF. Relying on morphological parameters of PF such as hypoechoic areas and thickening of plantar fascia is an unreliable standard as there are no anthropometric studies to standardize the values in various regions. Also, there are no standard protocols described to assess the thickness of plantar fascia as it even varies with the position of the great toe during assessment [9] or even with chronic diabetes mellitus [10]. Hansen et al. reported that the thickness of plantar fascia can also decrease over time in patients with PF and that at over 10-year follow-up, they can still have hypoechoic and thickened areas in 75% of asymptomatic individuals [11]. Hence, B-mode (Gray-scale) sonography has a role in differentiating PF from other differential conditions, but it is a weak tool for diagnosed cases of PF to assess or evaluate the severity of the condition or to analyze the recurrent conditions. 

Putz et al. carried out a pilot study on the utility of contrast-enhanced US and SWE in PF and found that both are useful [12]. Sonoelastography for PF was studied by Wu et al. who concluded that plantar fascia softens with age and in subjects with plantar fasciitis. This continued to be unreliable again as there was no validation with respect to age. Wu et al. studied changes in PF after shock wave therapy by hue histogram analysis and found it to be an easier tool to reflect color changes and have less inter-observer variability [13]. We feel that a small area analyzed as the ROI on a color histogram may not represent the actual sample. 

Gatz et al. were the first to study SWE in comparison with B-mode US in the utility of PF and evaluated the thickness of plantar fascia and Young's modulus to clinical scores [7]. Our study findings are in line with those of Gatz et al., where the affected side of plantar fascia is less elastic than the unaffected side. This can be due to degenerative changes in the fascia resulting in the breakdown of collagen, fibroblastic hypertrophy, and matrix degradation [14,15]. We have included only unilaterally affected subjects in our study compared to healthy individuals by Gatz et al., making it unique. However, a smaller sample size is our limitation. We did not take into account any potential correlations between comorbidities, gastrocnemius tightness, and existing foot and ankle deformities, which could have provided valuable insights into the interplay between these factors.

## Conclusions

We observed that SWE is a great additive tool to B-mode ultrasonography and in some cases, it is very helpful as it can even detect early PF with normal thickness of plantar fascia. This reliable tool, which is user-independent, can be used for monitoring efficacy of a treatment protocol and can be a reliable data tool to corelate functional and pain scores. More studies and a larger population series can also help in formulating a clinico-radiological classification system for PF, which can predict the condition and suggest treatment outcomes.
